# *Acrocomia aculeata* (Mbokaja) Kernel Oil Inhibits Herpes Simplex Virus 1 Replication and Promotes Cutaneous Wound Healing in Infected Mice

**DOI:** 10.3390/molecules31173092

**Published:** 2026-09-03

**Authors:** Ana Belén Villalba Aucejo, María Laura Correa Quevedo, María Paz Cáceres-Villalba, Gladys Azucena Romero Saldivar, Antonia Galeano, Juan Daniel Rivaldi Chavez, Patricia Langjahr, Pablo H. Sotelo

**Affiliations:** 1 Biotechnology Department, Facultad de Ciencias Químicas, Universidad Nacional de Asunción, San Lorenzo 111421, Paraguay; anavillalba800@gmail.com (A.B.V.A.); mcaceres@qui.una.py (M.P.C.-V.); plangjahr@qui.una.py (P.L.); 2 Industrial Aplications Department, Facultad de Ciencias Químicas, Universidad Nacional de Asunción, San Lorenzo 111421, Paraguay; lcorrea@qui.una.py (M.L.C.Q.); azucenaromero1992@gmail.com (G.A.R.S.); danielrivaldi@qui.una.py (J.D.R.C.); 3 Pharmacology Department, Facultad de Ciencias Químicas, Universidad Nacional de Asunción, San Lorenzo 111421, Paraguay; agaleano@qui.una.py

**Keywords:** *Acrocomia aculeata*, kernel oil, herpes simplex type 1, antiviral

## Abstract

**Background/Objectives**: Herpes simplex virus type 1 (HSV-1) is a global and prevalent pathogen, presenting significant clinical challenges because of its recurring infections, the development of drug resistance and severe clinical complications. This study evaluated the antiviral efficacy against HSV-1 of *Acrocomia aculeata* (*A. aculeata*) kernel oil, a Neotropical palm native to the Americas. **Methods**: The chemical profile of *A. aculeata* kernel oil was determined by gas chromatography with flame ionization detection (GC-FID). Antiviral activity was assessed using dose–response curves, time-of-addition assays, and quantification of intracellular viral genomes, viral gene transcripts, and IL-6 expression. *A. aculeata* kernel oil’s antiviral effect was also evaluated using an in vivo HSV-1 cutaneous infection model. **Results**: GC-FID analysis revealed lauric, oleic, and myristic acids as predominant components in the kernel oil. *A. aculeata* kernel oil exhibited potent antiviral activity against HSV-1. The oil inhibited HSV-1 early step post-entry, reducing the mRNA levels of the immediate-early genes ICP4 and ICP22, leading to the downregulation of early and late viral gene expression and intracellular viral genome. Furthermore, the oil suppressed IL-6 expression in infected cells. Importantly, *A. aculeata* kernel oil promoted the healing of cutaneous lesions in HSV-1-infected mice. **Conclusions**: These findings demonstrate that *A. aculeata* kernel oil is a promising candidate for developing novel antiviral and topical therapies against HSV-1.

## 1. Introduction

Herpes simplex virus type 1 (HSV-1) is an enveloped double-stranded DNA virus belonging to the Herpesviridae family [1], which affects approximately 66.6% of the global population [2]. Transmission typically occurs during childhood or adolescence, frequently manifesting as oral or facial lesions upon symptomatic onset [3]. Acyclovir, a nucleoside analog that disrupts viral DNA elongation and halts replication upon incorporation into newly synthesized viral genomes, remains the primary therapeutic intervention [4]. However, prolonged antiviral therapy in immunocompromised individuals, combined with impaired cell-mediated immunity, has driven the emergence of acyclovir-resistant strains. These resistant variants are associated with severe clinical complications, including herpetic pneumonia, esophagitis, and meningoencephalitis [5].

In addition to viral effects, the host immune response plays a crucial role in HSV-1 pathogenesis, with cytokines serving as key mediators of this process [6]. Among these, IL-6 and IL-8 are pivotal in driving lesion progression during recurrent herpes simplex labialis, a condition where tissue damage is primarily mediated by inflammatory responses rather than active viral replication [7].

In this regard, medium-chain fatty acids (MCFAs) and long-chain unsaturated fatty acids have been shown to inactivate HSV-1 by disrupting the lipid membrane of the viral envelope [8]. Additionally, some studies have reported that MCFAs inhibited the production of inflammatory cytokines, such as IL-6 and IL-8, thereby reducing inflammation and minimizing tissue damage caused by inflammatory responses [9]. These antiviral and immunomodulatory effects of plant-derived oils support their potential as promising candidates for the development of alternative antiviral therapies against HSV-1 [10,11].

*Acrocomia aculeata* (Jacq.) Lodd. ex Mart., also known as “Mbokaja” in Guaraní, is a palm species distributed across the neotropical and subtropical regions of America, spanning from Mexico to southern South America. Brazil and the eastern region of Paraguay exhibit the highest occurrence and phenotypic diversity of this palm [12,13]. Furthermore, *A. aculeata* is highly adapted to diverse climatic conditions and is recognized for its notable resilience to pests and diseases.

Among the various bioproducts obtained from *A. aculeata*, pulp and kernel oils hold the greatest commercial value. Pulp oil, which is rich in unsaturated fatty acids, is primarily utilized for biodiesel production, whereas kernel oil—enriched with saturated fatty acids such as lauric acid—is predominantly used in the cosmetics industry for soap and skincare formulations [14,15,16,17]. Beyond its industrial applications, *A. aculeata* oil exhibits significant therapeutic potential. Both pulp and kernel oils possess antioxidant activity, reducing reactive oxygen species (ROS) formation in HepG2, A549, IMR90, and Caco-2 cell lines [18]. Furthermore, these oils display chemopreventive properties, significantly reducing DNA damage and apoptosis induced by cyclophosphamide, and modulating immune responses in mice [19]. Previous studies have also demonstrated the anti-inflammatory, antifungal (against *Candida albicans*), and antibacterial activities of *A. aculeata* oils, which inhibit both sensitive and multidrug-resistant (MDR) strains of *Escherichia coli*, *Staphylococcus aureus*, *Pseudomonas aeruginosa*, and *Enterococcus faecalis* [20,21,22,23]. Conversely, the antiviral properties of *A. aculeata* oils remain completely unexplored.

Natural oils from various plants have gained attention for their broad-spectrum antiviral activities, particularly in the context of emerging resistance to conventional antivirals. Recent research has demonstrated the efficacy of plant-derived oils against HSV, influenza, and specific coronaviruses [24,25,26,27]. These oils contain fatty acids and complex mixtures of bioactive compounds capable of targeting multiple stages of the viral life cycle, thereby offering complementary or alternative therapeutic options for the management of viral diseases [28].

This study demonstrates the antiviral effectiveness of *Acrocomia aculeata* kernel oil against HSV-1, revealing its capacity to inhibit early-stage viral cycle and downregulate IL-6 expression in infected cells. Further, topical application of *A. aculeata* oil accelerated cutaneous injury recovery in an in vivo mouse model of infection. Taken together, these findings elucidate the therapeutic antiviral potential of *A. aculeata* kernel oil, supporting its development as a novel topical candidate against HSV-1.

## 2. Results

### 2.1. Quality Parameters and Fatty Acid Profile of A. aculeata Kernel Oil

**Figure 1:**
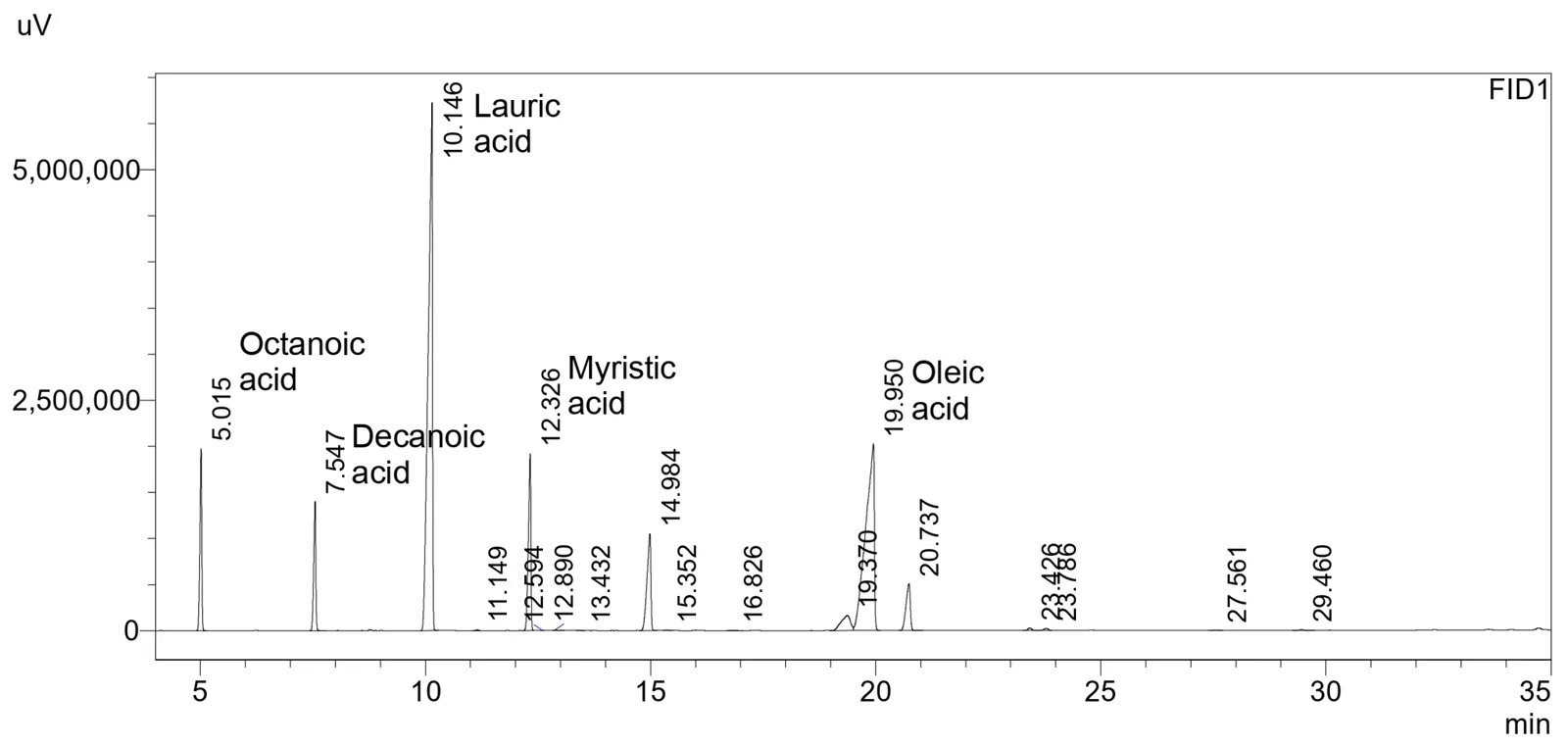
GC-FID chromatogram of *A. aculeata* kernel oil.

The chemical characterization of *A. aculeata* kernel oil included the iodine determination, saponification, peroxide, and acid values (Table 1). The fatty acid profile was determined quantitatively using gas chromatography with flame ionization detection (GC-FID), resulting in the identification of seventeen fatty acids through comparison with a reference fatty acid methyl ester (FAME) standard mix (Table 2 and Figure 1). Lauric acid (C12:0) was identified as the predominant component, constituting 39.44% of the total fatty acid composition, followed by oleic acid (C18:1, 28.92%) and myristic acid (C14:0, 7.81%). Other major fatty acids identified were palmitic acid (C16:0, 6.59%), octanoic acid (C8:0, 5.94%), and decanoic acid (C10:0, 4.66%).

### 2.2. Cell Viability and Antiviral Activity of A. aculeata Kernel Oil

**Figure 2:**
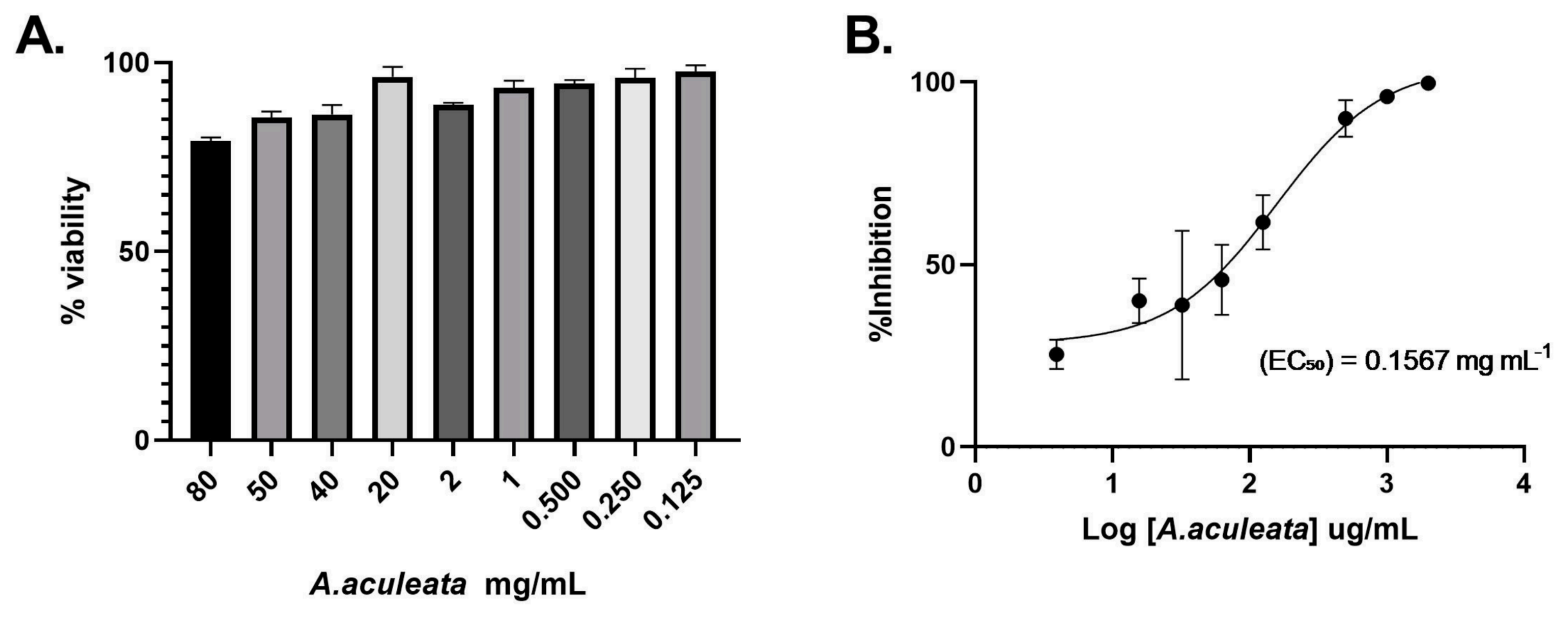
Cytotoxicity and antiviral effect of *A. aculeata* kernel oil. (**A**) Cytotoxicity of *A. aculeata* kernel oil. Vero cells were treated with the oil for 48 h. Cell viability was determined using the resazurin method. (**B**) Antiviral activity against HSV-1. Vero cells were infected at an MOI of 1.5, followed by the addition of serial dilutions of *A. aculeata* kernel oil. The levels of the viral genome in the supernatant were quantified using qPCR after 48 h. Data are expressed as mean ± SD, *n* = 3.

The cytotoxic effects of *A. aculeata* kernel oil on Vero cells were evaluated using the resazurin assay. At the highest soluble concentration tested (80 mg mL^−1^), the oil exhibited only 20% cytotoxicity. Due to the inherent lipophilicity and subsequent aqueous solubility limitations of the oil, a definitive 50% cytotoxic concentration (CC_50_) could not be determined within the soluble range (Figure 2A). Consequently, the maximum non-toxic concentration (MNTC), defined as the threshold causing no more than 10% cytotoxicity, was conservatively established at 2 mg mL^−1^ and selected for subsequent antiviral functional assays.

To evaluate the antiviral effect, serial dilutions of kernel oil were added to the infected cells after virus adsorption. The virus produced was quantified, and the half-maximal effective concentration (EC_50_) was 0.1567 mg mL^−1^ (Figure 2B). Based on the maximum tested soluble concentration, the selectivity index (SI) is estimated to be greater than 510.5.

### 2.3. A. aculeata Kernel Oil Inhibits HSV-1 Post-Entry by Reducing the mRNA Levels of Viral Genes

**Figure 3:**
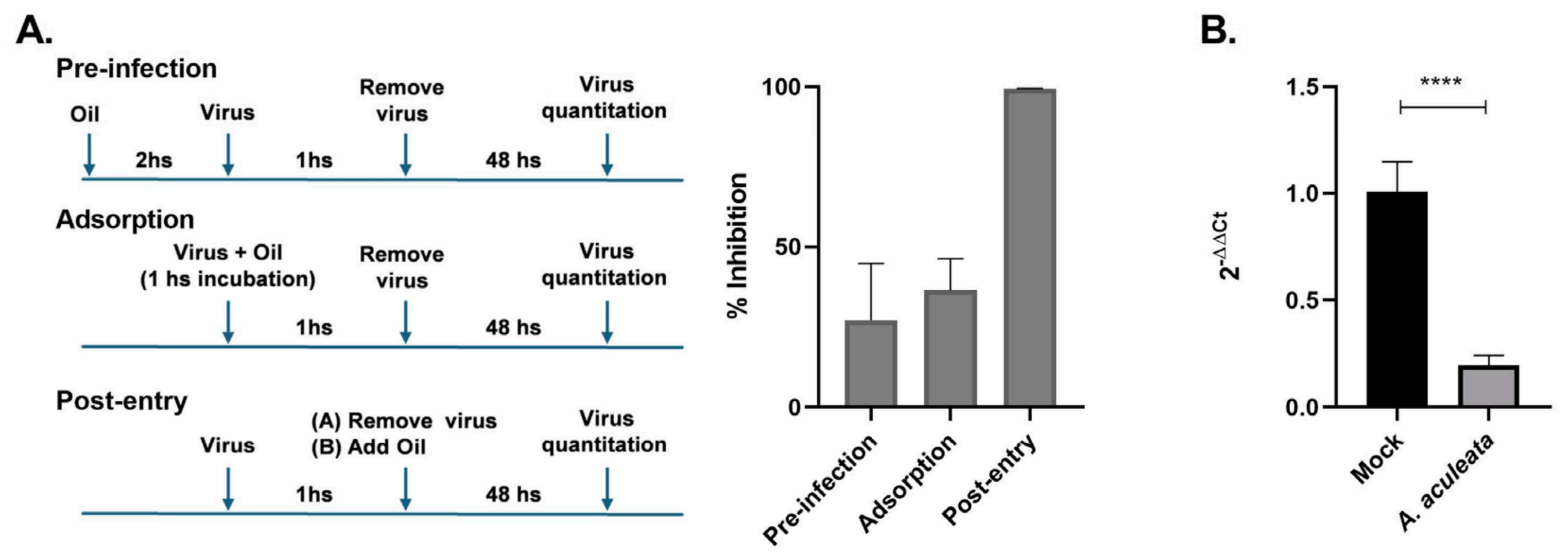
Time-of-addition assay and replication. (**A**) Time-of-addition assay. Vero cells were infected at an MOI of 1.5 and incubated with *A. aculeata* kernel oil at different times during infection. After 48 h, the viral genome production was quantified in the supernatant using qPCR. The percentage of viral inhibition was determined as the ratio of treated to untreated infected cells. (**B**) Vero cells were infected at an MOI of 5 and incubated in the presence or absence of *A. aculeata* kernel oil. After 8 h post-infection (hpi), intracellular genomes were quantified by RT-qPCR using the 2^−ΔΔCt^ method, using GAPDH as a normalizer gene. Mock = untreated cells; *A. aculeata* = *A. aculeata* kernel oil. Data are expressed as mean ± SD, *n* = 3 for each group. **** *p* < 0.0001.

To determine the stage of the viral replication cycle targeted by the oil, a time-of-addition assay was performed. As shown in Figure 3A, *A. aculeata* kernel oil exhibited antiviral activity predominantly during the post-entry condition, achieving 99% viral inhibition after the virus had entered the cell. In contrast, evaluating the induction of a cellular antiviral state through oil pre-treatment prior to infection yielded a lower effect, resulting in 27% viral inhibition. Similarly, treatment during the viral entry stage (adsorption) resulted in 36% viral inhibition. Since a significant antiviral effect was observed in the post-entry condition, the mechanism underlying HSV-1 inhibition was evaluated. The intracellular viral genomic DNA was quantified. Consistent with the time-of-addition assay results, a marked reduction in intracellular HSV-1 genome levels was observed in the oil-treated cells (Figure 3B).

**Figure 4:**
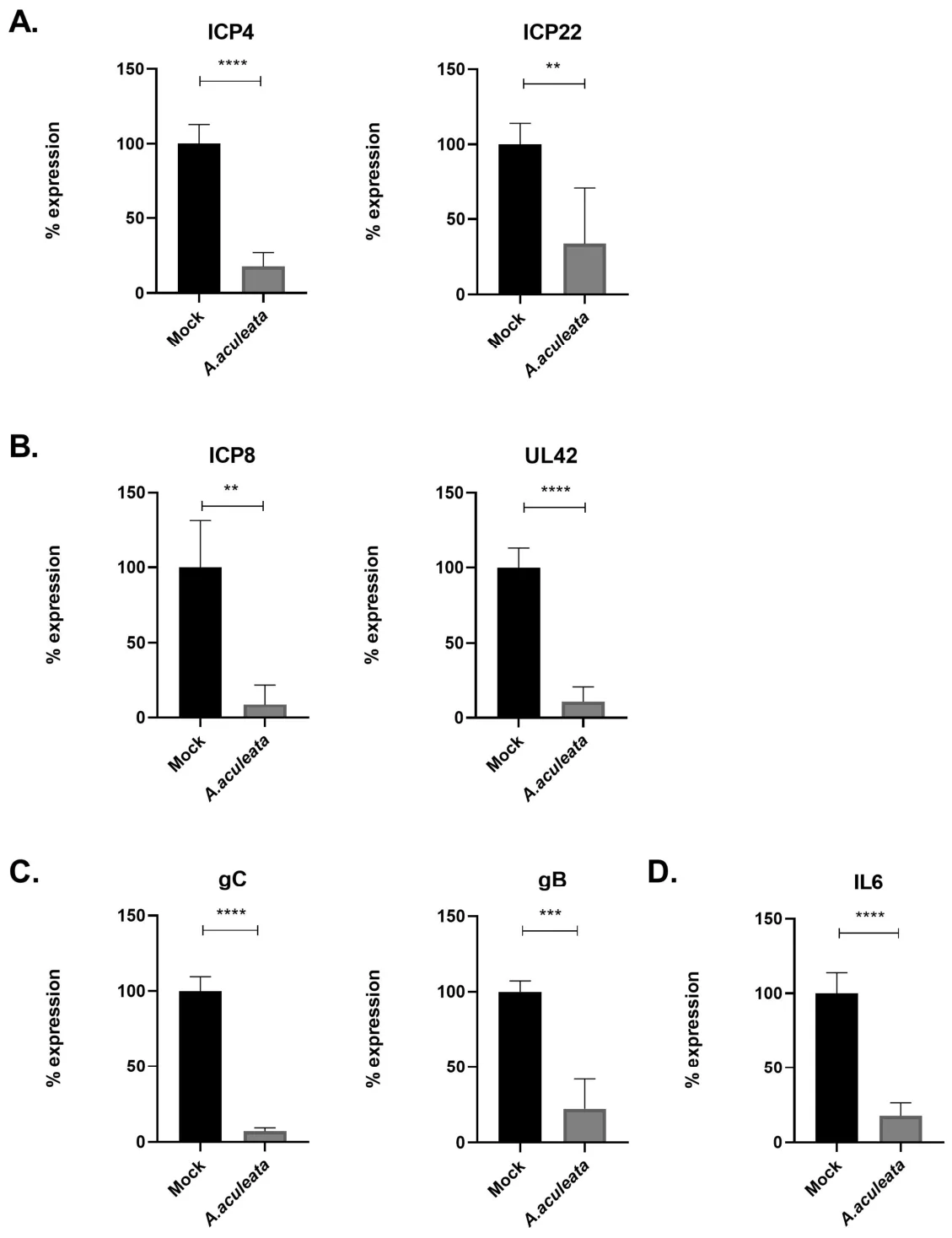
Effect of *A. aculeata* oil on viral cellular gene expression. (**A**–**C**) Expression of immediate-early (IE: ICP4, ICP22), early (E: ICP8, UL42), and late (L: gB, gC) HSV-1 genes. (**D**) Expression of the host inflammatory IL-6 gene. Vero cells were infected at an MOI of 5 and incubated in the presence or absence of *A. aculeata* kernel oil. After 8 h post-infection (hpi), mRNA was quantified by RT-qPCR using the 2^−ΔΔCt^ method, using GAPDH as a normalizer gene. Mock = untreated cells; *A. aculeata* = *A. aculeata* kernel oil. Data are expressed as mean ± SD, *n* = 3. ** *p* ≤ 0.01, *** *p* < 0.001 and **** *p* < 0.0001.

The decrease in intracellular viral genome levels may arise from direct interference with the viral replication machinery or the suppression of viral gene transcription. To investigate this, we analyzed the impact of *A. aculeata* kernel oil on viral mRNA production at 8 h post-infection (hpi). Specifically, we quantified the transcript levels of immediately early (IE: *ICP22*, *ICP4*), early (E: *ICP8*, *UL42*), and late (L: *gB*, *gC*) genes via RT-qPCR. *A. aculeata* kernel oil significantly reduced the mRNA levels of the IE (*ICP22*, *ICP4*), E, and L genes (Figure 4A–C). These findings suggest that *A. aculeata* kernel oil primarily exerts its antiviral effects during the early phase of the viral cycle by downregulating IE gene expression.

### 2.4. A. aculeata Kernel Oil Downregulates Virus-Induced IL-6 Expression

Considering the critical role of IL-6 in HSV-1 pathogenesis, we investigated the impact of *A. aculeata* kernel oil on IL-6 expression in infected cells. Our findings indicated that the oil led to a reduction in IL-6 mRNA levels at 8 hpi (Figure 4D).

### 2.5. A. aculeata Kernel Oil Reduced Lesion Severity in a Cutaneous HSV-1 Infection Model

The antiviral therapeutic efficacy of the oil was evaluated in a cutaneous HSV-1 infection mouse model. First, to establish the dermatological safety of the treatment, a skin irritation assay was conducted. Daily topical application of *A. aculeata* kernel oil for six consecutive days induced no visible signs of inflammation, erythema, or edema, confirming a favorable localized safety profile (Supplementary Figure S1).

**Figure 5:**
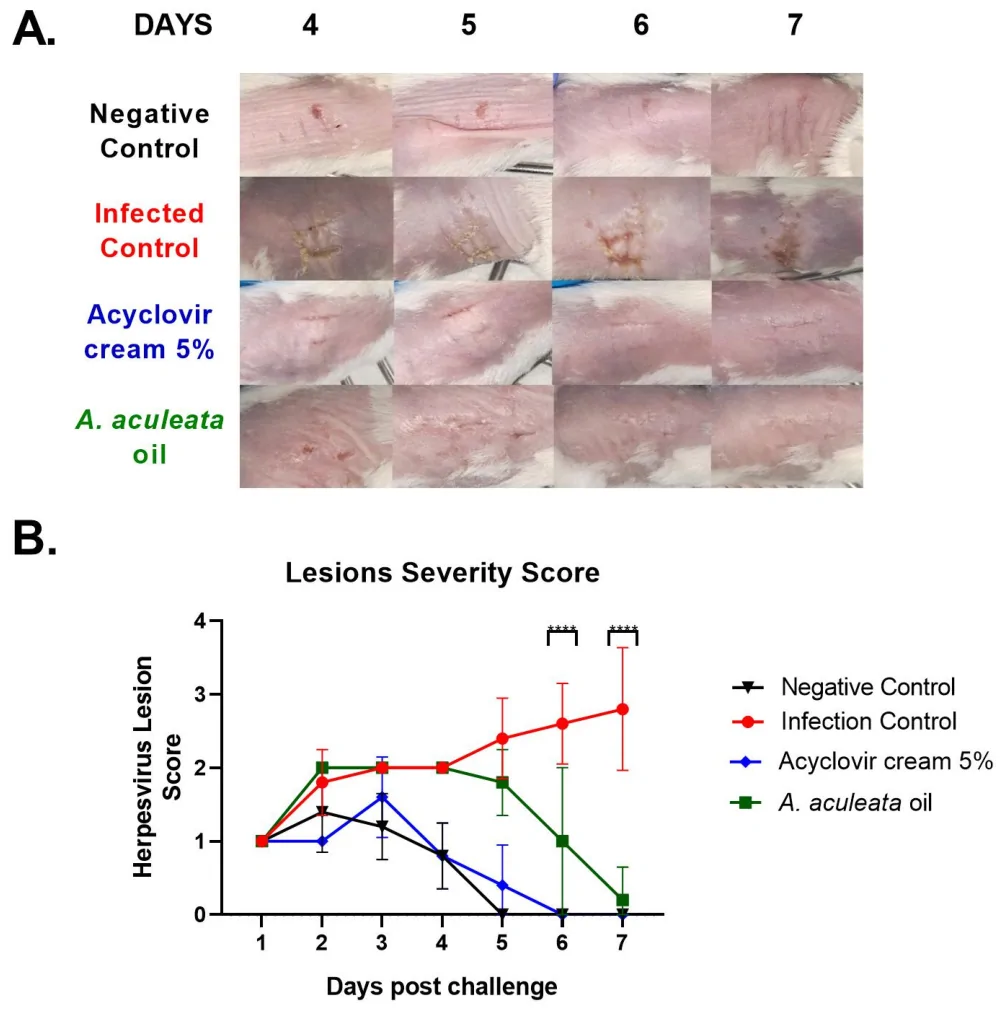
Therapeutic effect of *A. aculeata* oil in an HSV-1 skin infection model. Mice were infected with HSV-1 on the right flank skin and treated 24 h post-infection with 100 mg of *Acrocomia aculeata* oil once daily. Mock-infected mice, a commercially available 5% acyclovir cream, and infected untreated mice were included as control groups. (**A**) Representative images of skin lesions at days 4, 5, 6, and 7 post-infection with HSV-1. (**B**) Skin lesion scores were evaluated daily for 7 days. Data are presented as mean ± SEM of four mice per group (*n* = 5/group). Statistical analysis was performed using two-way ANOVA followed by Tukey’s multiple comparisons test. **** *p* < 0.0001.

Subsequently, the antiviral effect was evaluated. Untreated infected mice (positive control group) developed characteristic herpetic lesions by day 2 post-infection, peaking with a clinical score of 3.0 from day 5 through day 7. Topical treatment with *A. aculeata* kernel oil significantly reduced lesion severity compared to the untreated control group (*p* < 0.0001) (Figure 5). The oil promoted progressive healing, leading to significant lesion resolution by day 7. This therapeutic outcome was highly comparable to that of the standard treatment (5% acyclovir cream).

## 3. Discussion

This study demonstrates that *A. aculeata* kernel oil inhibits HSV-1 at the post-entry stage, as evidenced by the downregulation of IE gene expression. Additionally, the oil promoted lesion healing in an in vivo cutaneous HSV-1 infection model.

Establishing the physicochemical quality of *A. aculeata* kernel oil is fundamental to validating its prospective integration into therapeutic or cosmetic formulations. Given the absence of dedicated quality monographs for *A. aculeata* kernel oil, its physicochemical parameters were benchmarked against the Codex Alimentarius standards for African palm kernel oil (*Elaeis guineensis* Jacq.), another lauric acid-rich member of the *Arecaceae* family [29]. The saponification, peroxide, and free fatty acid values of *Acrocomia aculeata* kernel oil fall within the Codex limits for African palm kernel oil, supporting its suitability for cosmetic and pharmaceutical applications. Only the iodine value (32.0 ± 1.4 g I_2_ 100 g^−1^) exceeded the Codex range (14.1–21.0 g I_2_ 100 g^−1^), which may reflect species-specific or environmental influences on fatty acid desaturation [30]. Furthermore *A. aculeata* kernel oil is listed in the European Commission’s Cosmetic Ingredients (COSING) database (CAS No. 2331183-53-8). This regulatory inclusion validates its safety and quality profile, ensuring compliance with international standards and significantly streamlining its translational development into standardized dermatological, cosmetic, and pharmaceutical formulations.

*A. aculeata* oil exhibited minimal cytotoxicity in Vero cells, achieving only 20% cytotoxicity at the 80 mg/mL concentration. Consequently, its definitive CC_50_ value could not be determined due to the inherent aqueous solubility limitations of *A. aculeata* kernel oil (Figure 2). Despite these solubility constraints, the oil demonstrated a potent antiviral activity, with an EC_50_ value of 0.1567 mg mL^−1^. The SI, defined as the ratio of CC_50_ to EC_50_, serves as the margin between biological activity and toxicity. It is generally accepted that an antiviral product with an SI ≥ 10 is considered a candidate for further pre-clinical studies [31,32]. Based on the maximum non-toxic concentration tested, *A. aculeata* kernel oil exhibited an estimated SI > 510.5. Similar behavior has been observed for other lipophilic natural products with anti-HSV-1 activity, for which the CC_50_ could not be determined because of solubility limitations [33]. The high SI value, despite the low solubility-limited toxicity of the oil under the assay conditions, underscores a highly favorable safety margin between therapeutic efficacy and in vitro cell viability.

The low solubility of natural oils in aqueous media constitutes a critical limitation that restricts their dispersion, stability, and bioavailability, thereby compromising their biological efficacy. To overcome these barriers, novel drug delivery strategies have been developed. For instance, incorporating oily and hydrophobic components into three-dimensional polymeric matrices, such as multifunctional or reactive oxygen species (ROS)-responsive hydrogels, enables their controlled and localized release [34,35]. Furthermore, nanotechnology offers key platforms such as nanoemulsions, which encapsulate the lipid phase to increase the surface area, prevent phase separation, and significantly enhance apparent solubility, cellular permeation, and physicochemical stability [36,37,38]. Ultimately, these strategies could be implemented to develop novel *A. aculeata* formulations to enhance its overall efficacy profile.

The main fatty acid components of *A. aculeata* kernel oil were lauric (C12:0), myristic (C14:0) and oleic (C18:1), representing approximately 75% of total fatty acids (Figure 1). Previous studies have established that MCFA and long-chain unsaturated fatty acids, such as lauric and linoleic acids, can inactivate HSV-1 by disrupting the viral envelope [39,40]. However, the antiviral activity of *A. aculeata* oil against HSV-1 does not primarily result from its virucidal properties. When incubated directly with the virus, the oil demonstrated only 36% inhibition, suggesting that other mechanisms are likely to be more significant in their antiviral action (Figure 3).

Lauric acid, the primary constituent of *A. aculeata* kernel oil, has been documented to inhibit the replication of the enveloped arenavirus Junin (JUNV) through a non-virucidal mechanism that interferes with the insertion of viral glycoproteins into the plasma membrane during maturation [41]. However, while the potential impact of *A. aculeata* oil on viral assembly cannot be entirely ruled out, our findings strongly suggest that its primary antiviral target resides in the early stages of the replication cycle. Treatment with *A. aculeata* kernel oil led to the downregulation of the IE genes *ICP4* and *ICP22* (Figure 4A). These proteins have distinct viral regulation functions. ICP22 negatively influences RNA polymerase II processivity by decreasing serine 2 phosphorylation in its C-terminal domain, thereby modulating transcription elongation [42]. Conversely, ICP4 serves as the primary transcriptional activator of E and L viral genes [43]. Consequently, the downstream reduction in E and L viral gene expression upon *A. aculeata* kernel oil treatment can be primarily attributed to the downregulation of *ICP4* (Figure 4B,C). Notably, MCFAs, particularly octanoic acid, have been shown to inhibit other gamma herpesviruses. Previous studies demonstrated that MCFAs decrease Epstein–Barr virus activation and reduce protein levels of ORF50—the homolog of HSV-1 ICP4—in Kaposi’s sarcoma-associated herpesvirus, notably without affecting its transcription [44].

Cytokines are key mediators of the immunopathological responses triggered by HSV-1 infection [6]. Among these, IL-6 exerts pleiotropic effects and functions in both pro-inflammatory and anti-inflammatory roles [45]. The upregulation of IL-6 has been widely documented in various cell types following HSV-1 infection, including corneal epithelial cells, keratinocytes, fibroblasts, and leukocytes [46,47,48,49]. Furthermore, elevated IL-6 levels are strongly associated with severe inflammatory pathologies such as periodontitis and keratitis [50,51]. Moreover, IL-6 and IL-8 play pivotal roles in the progression of lesions during recurrent herpes simplex labialis, where the localized inflammatory response—rather than active viral replication—serves as the primary driver of tissue damage [7].

Given the previously reported anti-inflammatory properties of *A. aculeata* pulp oil [20,23], we evaluated the effect of kernel oil on virus-induced IL-6 expression. As shown in Figure 4D, *A. aculeata* kernel oil significantly reduced IL-6 mRNA levels in infected cells. Previous studies have established that IL-6 induction by HSV-1 is dependent on PKR activation but independent of the expression of VP16 or key immediate-early proteins such as ICP4, ICP8, and ICP27 [47]. Consequently, the observed reduction in IL-6 expression following *A. aculeata* treatment is likely not a secondary consequence of immediate-early gene inhibition but rather suggests a distinct, direct mechanism of action of the oil on the cellular inflammatory signaling cascade triggered by the infection. Notably, oleic acid attenuates the inflammatory response by inhibiting the NF-κB signaling pathway, thereby downregulating the expression and secretion of interleukin-6 (IL-6) [52,53]. Further research is required to delineate the specific cellular pathways through which the oil modulates the inflammatory response.

A fundamental prerequisite for any prospective topical formulation is the absolute absence of localized dermal toxicity. Our dermatological safety evaluation demonstrated that the daily application of 100 mg of *A. aculeata* kernel oil for six consecutive days induced no macroscopic signs of irritation, erythema, or edema. This high biocompatibility aligns with the historical ethnopharmacological use of this oil in folk medicine and is consistent with the dermal tolerance profiles reported for other therapeutic vegetable oils rich in medium-chain fatty acids, such as virgin coconut oil (*Cocos nucifera*) [54].

The mouse model of cutaneous herpes simplex infection is a well-established surrogate for evaluating topical antiviral therapeutics. The clinical progression of epidermal lesions and local inflammatory responses in this rodent model share key pathological features with human herpes labialis and cutaneous HSV infections [55]. In the cutaneous HSV-1 infection model, untreated infected mice developed characteristic herpetic lesions by day 2 post-infection, which progressed to a peak clinical score of 3.0 (characterized by localized inflammation, ulceration, and zosteriform lesions) from day 5 through day 7. Topical intervention with *A. aculeata* kernel oil significantly attenuated this pathological progression (Figure 5), successfully preventing the transition into severe, coalescent zosteriform lesions and promoting progressive re-epithelialization that culminated in substantial clinical resolution by day 7. Notably, the oil’s therapeutic effect was similar to that of the standard acyclovir treatment.

Beyond individual fatty acid activities, the complex composition of *A. aculeata* kernel oil acids strongly suggests a multi-target mechanism. While MCFA like octanoic acid may penetrate cellular membranes to interfere with early intracellular events and viral transcription, the high content of monounsaturated oleic acid plays a complementary role by enhancing membrane permeability and exerting intrinsic anti-inflammatory and tissue-regenerative effects [44,56]. Together, this concerted lipid matrix acts to simultaneously suppress HSV-1 replication and accelerate cutaneous lesion recovery.

## 4. Materials and Methods

### 4.1. Virus and Cells

Vero cells from ATCC (CCL-81) cells were cultured in DMEM (GIBCO) supplemented with 7.5% fetal bovine serum (FBS; GIBCO). The HSV-1 strain F was propagated in Vero cells, and the viral stocks were quantified using a plaque assay.

### 4.2. Plant Material and Kernel Oil Extraction

The fruits of A. aculeata (Jacq.) Lodd. ex Mart were harvested at the ripe stage in Yaguarón, located in the Paraguarí department of Paraguay. The morphological features, component characteristics, and ecological distribution of the *Acrocomia aculeata* (Jacq.) Lodd. ex Mart are documented in the official logs of the Herbarium of the Faculty of Chemical Sciences at the National University of Asunción, voucher N°: Degen, R. 4490 (FCQ). Additionally, the specific morphoanatomical characteristics and regional distribution of *A. aculeata* are described by González et al. [57]. Fruits exhibiting mechanical damage or signs of microbial contamination were discarded. The fruits were subsequently dried using a silo-type dryer until they achieved equilibrium moisture content, thereby facilitating their handling during oil extraction. The fruits were then manually peeled to separate the kernels from the other components.

The kernel oil was extracted using a screw press, and the extracted oil was centrifuged at room temperature for 5 min at 4000 rpm to facilitate the separation of fine cake particles. The oil was then stored under refrigeration until further analysis.

### 4.3. Chemical Characterization

The chemical properties of the *A. aculeata* kernel oil were assessed according to the standard protocols established by the American Oil Chemists’ Society (AOCS). The iodine value was determined as the amount of iodine absorbed by 100 g of the sample, following the AOCS Cd 1c-85 method. The saponification number was defined using the AOCS Cd 3a-94 method, as the quantity, in milligrams, of potassium hydroxide required to saponify 1 g of oil. The free fatty acid content, expressed as oleic acid, was determined according to the AOCS method, Ca 5a-40. The peroxide value, expressed in milliequivalents of active oxygen per kilogram of oil, was measured using the Cd 8b-90 method stipulated by the AOCS.

### 4.4. Methyl Esters and Fatty Acid Analysis

The fatty acid composition was assessed following the transesterification of samples into fatty acid methyl esters (FAMEs) using potassium hydroxide in methanol, according to the AOCS Ce 2-66 method. FAMEs were subsequently analyzed using a gas chromatograph (GC-2010 Plus, Shimadzu, Kyoto, Japan) equipped with a flame ionization detector (FID) and a Stabilwax^®^ capillary column (30 m × 0.32 mm i.d, 0.25 μm film thickness; Restek, Bellefonte, PA, USA). High-purity nitrogen (99%) was used as the carrier gas at a total flow rate of 42 mL/min. Injector and detector temperatures were both maintained at 250 °C. Samples were injected using a split injection mode.

The oven temperature program was initiated at 60 °C (held for 0.5 min), increased at 10 °C/min to 180 °C (held for 5 min), ramped at 5 °C/min to 215 °C, then at 1 °C/min to 215 °C, and finally at 5 °C/min to 240 °C (held for 16 min), resulting in a total run time of approximately 50 min. Peak identification was achieved by comparing retention times with a standard reference mixture of FAMEs (F.A.M.E. Mix C8–C22, Sigma-Aldrich, St. Louis, MO, USA). Quantification was performed based on relative peak area normalization and expressed as a percentage of the total detected peak areas.

### 4.5. Cytotoxicity Assay

The cytotoxicity assay was performed as previously described [58]. Vero cells were seeded in 96-well plates at a density of 1 × 10^4^ cells per well in DMEM with 2% FBS, with or without the oil. Considering the solubility of *A. aculeata* oil, microscopic evaluation of treated cell monolayers across all evaluated concentrations confirmed intact cell morphology and the absence of insoluble lipid microdroplets. After 48 h, cell DMEM with 2% FBS containing resazurin was added to a final concentration of 0.0015%. Following a 3 h incubation period, absorbance was measured at 570 and 630 nm.

### 4.6. Antiviral Assays

#### 4.6.1. Dose–Response Curve

Vero cells were plated in 96-well plates and infected with the virus at an MOI of 1.5 in DMEM with 2% FBS. After one hour, the virus was removed, and DMEM with 2% FBS, with or without *A. aculeata* kernel oil, was added. Each experiment was performed in triplicates. Infected cells without treatment served as positive controls, and uninfected cells served as negative controls. At 48 h post infection (hpi), supernatants were collected and analyzed by qPCR.

#### 4.6.2. Virus Quantification

All qPCR reactions were performed as described by Gabaglio et al. [59]. Each reaction mixture contained 5 μL of 2X SYBR^®^ Green Master Mix (Bio-Rad Laboratories, Hercules, CA, USA), 500 pM of ICP0 FOR and ICP0 REV primers (Table 3), and 1 μL of the sample. Amplification was conducted using a StepOnePlus™ thermocycler (Thermo Thermo Fisher Scientific, Waltham, MA, USA.) employing a two-step program: initial denaturation at 95 °C for 5 min, followed by 35 cycles of 15 s at 95 °C and 30 s at 60 °C, ending with a melting curve analysis. Viral genome copies (VGCs) were calculated using a standard calibration curve.

#### 4.6.3. Time of Addition Assay

The assay was conducted under three distinct experimental conditions, as previously described [58]. For the pre-infection assay, cells were treated with *A. aculeata* kernel oil (2000 μg mL^−1^) for 2 h at 37 °C, washed with PBS, and infected for 1 h at 37 °C. For the adsorption assay, *A. aculeata* kernel oil (2000 μg mL^−1^) was mixed with the viral inoculum and incubated for 1 h at room temperature before being added to the cells for infection for 1 h at 37 °C. For the post-entry assay, cells were infected for 1 h at 37 °C and 2000 μg mL^−1^ *A. aculeata* kernel oil was added. In all assays, after infection, cells were washed with PBS, incubated for 48 h, and viral production was quantified by qPCR. Uninfected cells served as negative controls, and infected but untreated cells served as a normalizer.

#### 4.6.4. RNA and gDNA Analysis

Vero cells were plated in six-well plates at a density of 2 × 10^5^ cells per well. The following day, the cells were washed and infected with HSV-1 at an MOI of 5. After 1h, the viral inoculum was removed, followed by the addition of DMEM containing 2% FBS and *A. aculeata* kernel oil at a concentration of 2000 μg mL^−1^. At 8 hpi, RNA and viral DNA were extracted using TRIzol^®^ (Invitrogen, Thermo Fisher Scientific, Carlsbad, CA, USA.) according to the manufacturer’s instructions. RNA was treated with RQ1 RNase-Free DNase (Promega Corporation, Madison, WI, USA.) at 37 °C for 40 min, followed by inactivation at 65 °C for 10 min. RNA concentration was measured at 260/280 nm using a Multiskan™ FC Microplate Photometer with a μDrop™ Plate (Thermo Fisher Scientific, Waltham, MA, USA.). Two micrograms of RNA were used for cDNA synthesis using the MMLV Reverse Transcriptase kit (Promega), following the manufacturer’s instructions. mRNA levels and intracellular viral genome were quantified by qPCR, using the primers listed in Table 3, and data were analyzed using the 2^−ΔΔCt^ method, with GAPDH as the endogenous control. Untreated infected cells served as controls.

### 4.7. In Vivo Assays

#### 4.7.1. Animals

Female BALB/c mice (5–7 weeks old, 18–25 g) from the Bioterium Prof. Dra. Yenny Montalbetti of Chemical Sciences School, National University of Asunción, were used. Animals were assigned to experimental groups using a randomized block design based on baseline body weight to ensure uniform distribution across groups prior to infection. Potential confounding factors were controlled by maintaining strictly standardized environmental conditions (temperature at 22 ± 2 °C, 12 h light/dark cycle) and housing animals under identical conditions in individually ventilated cages with ad libitum access to food and water.

#### 4.7.2. Skin Irritation Test

To assess the potential dermal irritation of the *A. aculeata* kernel oil, a skin irritation assay was performed using four mice (*n* = 4 mice) and a non-treated control. The animals were anesthetized by intraperitoneal administration of ketamine at 75 mg/kg and xylazine at 5 mg/kg. After anesthesia, the hair on the left flank was carefully removed using a razor blade, exposing an area of approximately 4 ± 0.5 cm^2^.

The depilated area was treated with 100 mg of *A. aculeata* kernel oil once daily for six days, during which skin reactions were monitored. All animals were examined for signs of erythema and edema. Dermal reactions were graded according to the Organization for Economic Co-operation and Development (OECD) rating system [60]. All animals survived the study period, and no data points were excluded from the analysis.

#### 4.7.3. Evaluation of the Antiviral Activity in Mice

The cutaneous HSV-1 infection model was adapted from Bhutta et al. [61]. Sample size was determined using the Resource Equation Method (E = N − k). For our experimental design comprising 4 groups with *n* = 5 female BALB/c mice per group (total *N* = 20), the calculated error degrees of freedom (E = 16) falls within the recommended acceptable range [62]. Mice were randomly assigned to treatment groups, and inclusion and exclusion criteria were established a priori. Animals were included in the study if they were healthy female BALB/c mice and successfully developed characteristic cutaneous herpes simplex lesions following viral inoculation. Humane endpoints (e.g., severe body weight loss > 20% or systemic signs of severe distress) were defined prior to the study as explicit exclusion criteria. Because all infected animals developed characteristic lesions and none met any humane exclusion criteria, no animals or data points were excluded from the final analysis. Under intraperitoneal anesthesia (75 mg/kg ketamine and 5 mg/kg xylazine), the right flank (4 ± 0.5 cm^2^) was shaved. The exposed area was superficially scarified with a 26-gauge needle to induce slight excoriation, ensuring epidermal disruption without bleeding. Each mouse was then inoculated with 50 µL of DMEM containing 7 × 10^7^ PFU of HSV-1, which was gently spread over the scarified area until complete absorption.

After 24 h post-infection, 100 mg of either *A. aculeata* kernel oil or a commercial 5% acyclovir cream (Saval, Chile) was topically applied to the infection site once daily for 7 consecutive days. Two control groups were included: a scarified, non-infected group (negative control) and an infected, untreated group (positive control). Lesion severity was monitored daily using the following clinical scoring system: 0—no visible signs of infection; 1—pre-lesions: yellowish wounds with signs of inflammation; 2—vesicle or ulcer formation at the inoculation site; 3—small zosteriform rash lesions outside the inoculation site (reddish-yellowish ulcers, inflamed with or without pus); 4—larger, expanded zosteriform lesions; 5—confluent band of zosteriform lesions; 6—hind limb paralysis (or other neurological signs such as disorientation, limb discoordination); 7—death. To eliminate evaluation bias, a double-blinded scoring procedure was implemented: two independent evaluators assessed and scored the cutaneous lesions daily without knowledge of the treatment groups, as cages were coded with non-descriptive arbitrary numbers. Photographs of all animals from every experimental group are provided in the Supplementary Materials (Figure S2).

### 4.8. Statistical Analysis

The results are expressed as the mean ± standard deviation (SD) from at least three independent experiments. Differences within each group were analyzed using *t*-test or two-way ANOVA followed by Tukey’s multiple comparisons test with GraphPad Prism 8.01 (GraphPad Software Inc., Boston, MA, USA). Statistical significance was defined as follows: * *p* ≤ 0.05, ** *p* ≤ 0.01, *** *p* ≤ 0.001, and **** *p* ≤ 0.0001.

## 5. Conclusions

In conclusion, this study establishes *A. aculeata* kernel oil as a highly promising natural bioproduct for the topical management of HSV-1 infections. The therapeutic potential lies in a multi-level mechanism of action, effectively impairing post-entry viral replication by downregulating the key immediate-early genes ICP4 and ICP22 while simultaneously suppressing virus-induced IL-6 expression. Crucially, its preclinical efficacy in promoting lesion healing, coupled with a good dermal safety profile and formal COSING registration, supports its development as an active ingredient in standardized dermatological and pharmaceutical formulations. Nevertheless, successful clinical translation will require addressing key future challenges, including overcoming the low aqueous solubility and formulation constraints of the raw oil through advanced topical delivery systems, as well as conducting clinical trials to validate its efficacy and safety in human populations.

## Figures and Tables

**Table 1:** Chemical properties of *A. aculeata* kernel oil.

Chemical Properties	Value
Iodine value (g I2 100 g^−1^)	32.0 ± 1.4
Saponification value (mg KOH g^−1^)	242 ± 5.6
Peroxide value (mEq Kg^−1^)	2.1 ± 0.23
Free fatty acid (% as lauric acid)	1.38 ± 0.01

**Table 2:** Fatty acid composition of *A. aculeata* kernel oil.

Fatty Acid	Retention Time (min)	%
Octanoic acid (C8:0)	5.015	5.94
Decanoic acid (C10:0)	7.547	4.663
Lauric acid (C12:0)	10.146	39.444
Tridecanoic acid (C13:0)	11.149	0.028
Myristic acid (C14:0)	12.326	7.807
Myristoleic acid (C14:1)	12.594/12.890	0.032
Pentadecenoic acid (C15:1)	13.432	0.016
Palmitic acid (C16:0)	14.984	6.586
Palmitoleic acid (C16:1)	15.352	0.031
Stearic acid (C18:0)	16.826	0.025
Elaidic acid (C18:1)	19.370	2.486
Oleic acid (C18:1)	19.950	28.92
Linoleic acid (C18:2)	20.737	3.578
cis-11-Eicosenoic acid (C20:1)	23.426	0.168
Linolenic acid (C18:3)	23.786	0.172
Behenic acid (C22:0)	27.561	0.039
Erucic acid (C22:1)	29.460	0.064

**Table 3:** List of primers used in this study.

Gene	Primer Sequence	Tm	Size (bp)
RL2: ICP0	FOR: 5′-GTCGCCTTACGTGAACAAGAC-3′ REV: 5′-GTCGCCATGTTTCCCGTCTG-3′	59.5 61.6	114
RS1: ICP4	FOR: 5′-CGGTGATGAAGGAGCTGCTGTTGC-3′ REV: 5′-CTGATCACGCGGCTGCTGTACA-3′	66.5 65.5	145
UL29: ICP8	FOR: 5′-CATCAGCTGCTCCACCTCGCG-3′ REV: 5′-GCAGTACGTGGACCAGGCGGT-3′	65.9 66.9	130
US1: ICP22	FOR: 5′-ATGCAATGCTACGGCGCTCGGT-3′ REV: 5′-ACAGCTGATTGATACACTGGCGC-3′	67.6 63.6	99
UL42	FOR: 5′-ACGTCCGACGGCGAGG-3′ REV: 5′-CAGGCGCAACTGAACGTC-3′	61.6 59.5	111
UL27: Glycoprotein B (gB)	FOR: 5′-TACTGCGGCTGGCCCACCTTG-3′ REV: 5′-GCTCTCGCGCGTGGACCTG-3′	67.3 66.1	114
UL44: Glycoprotein C (gC)	FOR: 5′-GAGGAGGTCCTGACGAACATCACC-3′ REV: 5′-CCGGTGACAGAATACAACGGAGG-3′	64.5 63.1	140
GADPH (African green monkey kidney)	FOR: 5′-GAAGGTGAAGGTCGGAGT-3′ REV: 5′-CATGGGTGGAATCATATTGGAA-3′	55.9 57.9	155
IL-6 (African green monkey kidney)	FOR: 5′-TCTCCACAAGCGCCTTCG-3′ REV: 5′-CTCAGGGCTGAGATGCCG-3′	60.1 59.9	193

## Data Availability

The data presented in this study are available in the article. Additional raw data are available from the corresponding author upon reasonable request.

## Supplementary Materials

The following supporting information can be downloaded at: https://www.mdpi.com/article/10.3390/molecules31173092/s1, Figure S1: Skin irritation test. Representative images of female BALB/c mice topically treated with *A. aculeata* oil (*n* = 4/group) or untreated (negative control group). Animals received a daily application of 100 mg of the oil for 6 consecutive days. Signs of skin irritation and inflammation were evaluated 24 h post-treatment and monitored daily for the study period. Figure S2: Representative images of BALB/c mice in the cutaneous HSV-1 infection model. Female BALB/c mice were infected with HSV-1 and topically treated once daily for 7 consecutive days with *A. aculeata* kernel oil 100mg/animal, 5% acyclovir cream or untreated (infection control group). The negative control corresponds to uninfected and untreated mice. Lesion development was monitored and evaluated daily for 7 days.

## Abbreviations

The following abbreviations are used in this manuscript:

HSV-1	Herpes simplex type 1
IL-6	Interleukin-6
GC-FID	Gas Chromatography with Flame Ionization Detector
DNA	Deoxyribonucleic acid
mRNA	Messenger ribonucleic acid
ICP4	Infectious Cell Protein-4
ICP22	Infectious Cell Protein-22
IL-8	Interleukin-8
MCFAs	Medium-chain fatty acids
ROS	Reactive oxygen species
MDR	Multidrug-resistant
FAME	Fatty acid methyl esters
MNTC	Maximum non-toxic concentration
qPCR	Quantitative polymerase chain reaction
EC_50_	Half-maximal effective concentration
SI	Selectivity index
MOI	Multiplicity of Infection
gDNA	Genomic deoxyribonucleic acid
hpi	hours post infection
IE	Immediate early genes
E	Early genes
L	Late genes
GAPDH	Glycerinaldehyd-3-phosphat-Dehydrogenase
