# Supplementary material for: Acrocomia aculeata (Mbokaja) Kernel Oil Inhibits Herpes Simplex Virus 1 Replication and Promotes Cutaneous Wound Healing in Infected Mice
Source: Molecules. 2026 Sep 3;31(17):3092. doi: 10.3390/molecules31173092 (PMC13567627; doi:10.3390/molecules31173092)
Supplement: Supplementary file 1 [file molecules-31-03092-s001.zip › molecules-4503510-supplementary.pdf]

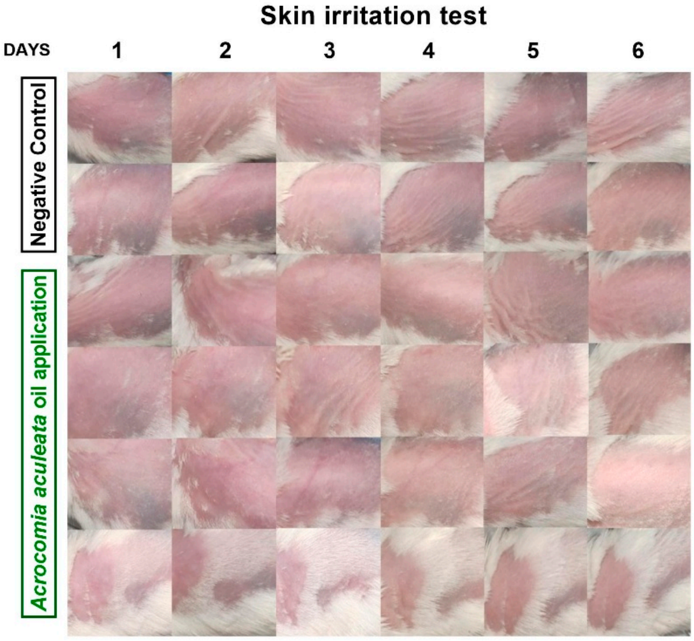

Figure S1. Skin irritation test. Representative images of female BALB/c mice topically treated with *A. aculeata* oil (n = 4/group) or untreated (negative control group). Animals received a daily application of 100 mg of the oil for 6 consecutive days. Signs of skin irritation and inflammation were evaluated 24 h post-treatment and monitored daily for the study period.

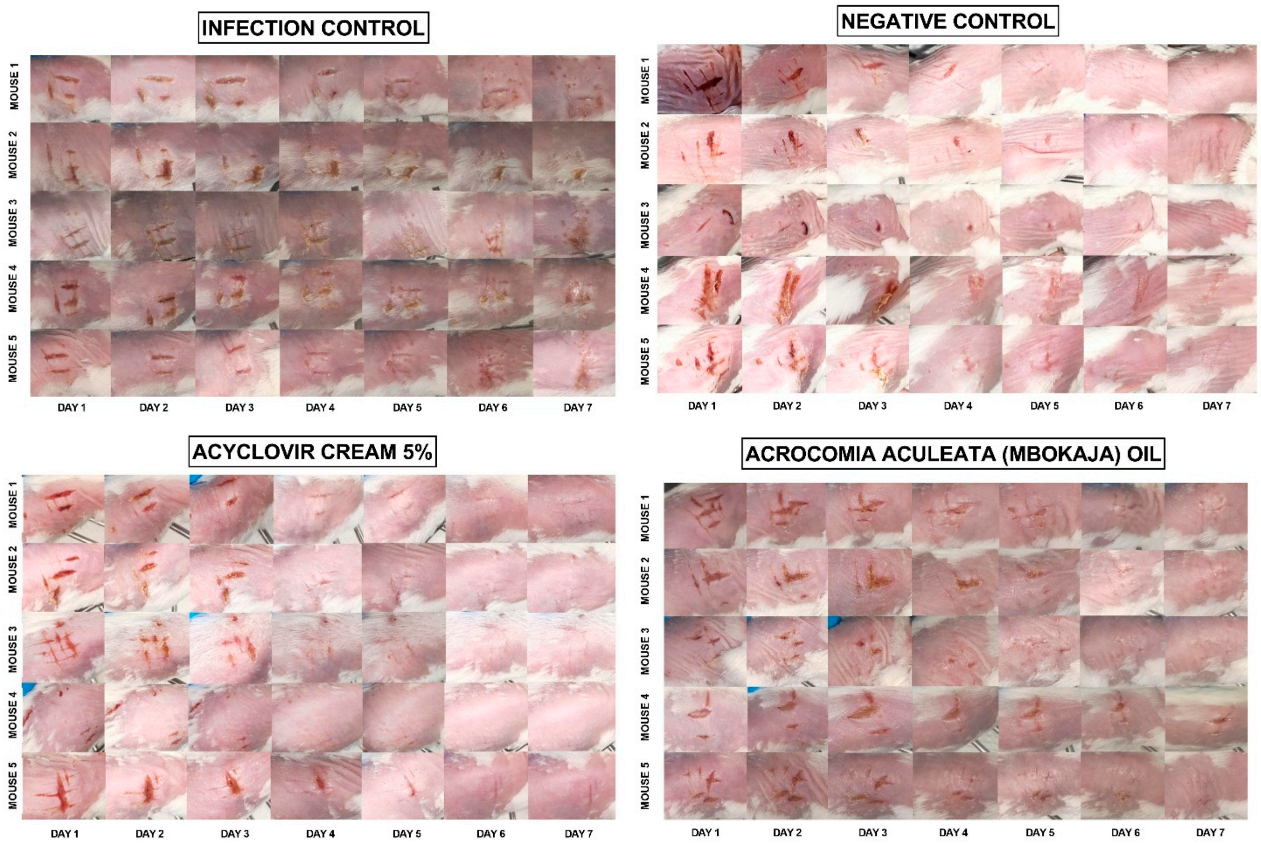

Figure S2. Representative images of BALB/c mice in the cutaneous HSV-1 infection model. Female BALB/c mice were infected with HSV-1 and topically treated once daily for 7 consecutive days with *A. aculeata* kernel oil 100mg/animal, 5% acyclovir cream or untreated (infection control group). The negative control corresponds to uninfected and untreated mice. Lesion development was monitored and evaluated daily for 7 days.
